# Rare Presentation of Marginal Papular Acrokeratoderma: A Case Report and Literature Review of Costa’s Acrokeratoelastoidosis of the Hand

**DOI:** 10.1155/crdm/8760488

**Published:** 2026-10-12

**Authors:** Ricardo Cid-Puente, Paulina Vázquez-Arroyo, Karla-Yareli Saucedo-Martínez, Angelica-Beatriz Rodríguez-Baca

**Affiliations:** ^1^ Department of Immunology Universidad Autónoma de Zacatecas Zacatecas Mexico uaz.edu.mx; ^2^ Dermatology Private Practice Servicios de Salud de Zacatecas Zacatecas Mexico; ^3^ General Practitioner Private Practice Servicios de Salud de Zacatecas Zacatecas Mexico; ^4^ Pathology Private Practice Servicios de Salud de Zacatecas Zacatecas Mexico

**Keywords:** acrokeratoelastoidosis, elastic fibers, genodermatosis, keratotic papules, rare dermatological condition

## Abstract

Acrokeratoelastoidosis (AKE) is a rare benign genodermatosis characterized by keratotic papules on the lateral aspects of hands and feet. We present a case of a 25‐year‐old woman with a 3‐year history of asymptomatic yellowish papules forming a linear plaque on the ulnar margin of her left hand. Previous treatments with topical acids and cryotherapy proved ineffective. Histopathological examination revealed hyperkeratosis, psoriasiform acanthosis, and decreased elastic fibers, confirmed by special stains. Treatment with high‐potency steroids and urea cream showed modest improvement. This case highlights the importance of considering AKE in the differential diagnosis of keratotic acral lesions and demonstrates the current therapeutic limitations in managing this uncommon condition.

## 1. Introduction

Acrokeratoelastoidosis (AKE) is a rare benign genetic skin disorder characterized by distinctive clinical features: firm, whitish, keratotic papules that develop asymptomatically on the lateral aspects of hands and feet [1]. According to the standard classification systems for palmoplantar keratodermas (PPK), this condition can be categorized based on its phenotypic expression; our case represents a sporadic, noncongenital, localized, and nonepidermolytic variant. Due to its uncommon occurrence in the general population, the precise incidence remains undetermined. A hypothesized mechanism for the condition’s pathophysiology involves abnormal secretion or production of elastic material by dermal fibroblasts. While this may provide a proposed explanation for the characteristic abnormalities of elastic fibers seen histologically, it does not fully explain the epidermal hyperkeratosis [2].

The diagnosis of AKE relies on a comprehensive evaluation of both clinical presentation and histopathological findings. Despite advances in dermatological treatments, there is currently no definitive cure for this condition. Various therapeutic approaches, including both topical and systemic treatments, have been attempted, though their effectiveness varies significantly among patients [1, 3]. This report presents a detailed case study of this rare dermatological condition, offering insights into its clinical manifestations, histopathological characteristics, and current treatment options, contributing to the broader understanding of this uncommon disorder.

## 2. Case Report

A 25‐year‐old woman presented to the dermatology department with no significant personal or family medical history. She had been experiencing asymptomatic dermatosis for 3 years and 8 months. The condition was localized on the ulnar margin of her left hand, extending to the ulnar aspect of her fifth digit. The lesion manifested as a linear plaque characterized by confluent yellowish papules, presenting a distinctive keratotic “cobblestone‐like” appearance (Figure 1). Prior to consultation, she had undergone multiple unsuccessful treatments, including topical applications of salicylic acid and lactic acid, as well as cryotherapy, all of which failed to produce any improvement.

**FIGURE 1:**
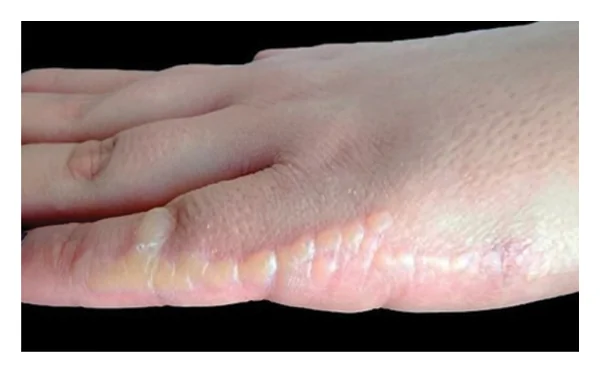
Linear confluent yellowish papules forming a cobblestone‐like plaque on the ulnar margin of the left hand and fifth digit.

Given the persistent nature of the condition, an incisional biopsy of the plaque was performed. The histopathological examination revealed several key findings: massive hyperkeratosis, regular psoriasiform acanthosis with hypergranulosis, and a scarce superficial perivascular infiltrate. The dermis showed slightly edematous collagen fibers and a mild reduction in elastic fibers (Figure 2). To confirm the diagnosis, special staining techniques were employed—both Verhoeff–van Gieson and Masson’s trichrome stains were performed, yielding positive results that supported the diagnosis of AKE (Figure 3). While electron microscopy (EM) would be valuable to further visualize the loss of fibers, it was not performed in this case.

**FIGURE 2:**
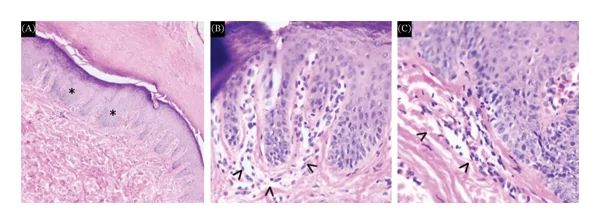
Histopathological findings under hematoxylin and eosin staining. (A) Massive acanthosis (black asterisk) (10*x* magnification). (B and C) Decreased collagen fibers with mild edematous changes in the dermis (black arrowhead) (40*x* magnification).

**FIGURE 3:**
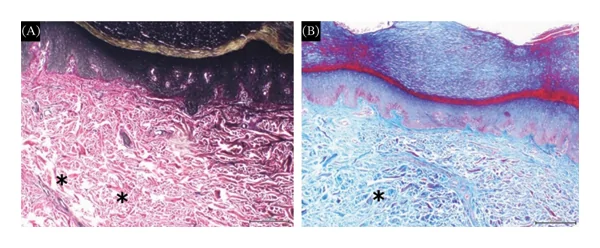
Histopathological findings showing decreased elastic fibers and elastorrhexis (black asterisk). (A) Verhoeff–van Gieson stain (10*x* magnification). (B) Masson’s trichrome stain (10*x* magnification).

Based on clinical and histopathological findings, the patient was started on a topical regimen consisting of high‐potency therapy with calcipotriol/betamethasone dipropionate applied twice daily, in combination with 50% urea cream. After 6 months of treatment, the patient showed modest clinical improvement.

## 3. Discussion

Costa’s AKE, also called inverse papular acrokeratosis, is a rare genodermatosis with an autosomal dominant pattern with incomplete penetrance [1]. It was first described in 1953 by Dr. Oswaldo Costa in Brazil [4]. It belongs to the group of marginal papular acrokeratodermas that includes focal acral hyperkeratosis (FAH), acral keratoelastoidosis of the hands, acrokeratosis verruciformis of Hopf, punctate PPK, and marginal collagenosis of the hands [3].

In 1994, Rongioletti et al. established a classification system dividing these pathologies into hereditary (with or without elastorrhexis) and acquired forms [5].

AKE is fundamentally regarded as a hereditary disorder; however, sporadic cases occur without a recognized familial history. In our patient’s case, no family history was identified, making this a sporadic presentation. It is important to distinguish AKE from acquired disorders within the broader group of marginal papular keratodermas that can clinically resemble AKE. For example, keratoelastoidosis marginalis (KEM) is an acquired keratoderma generally occurring in older adults and associated with chronic sun exposure and repetitive trauma. The distinction between hereditary AKE and acquired marginal keratodermas is crucial for accurate diagnosis [6, 7].

The exact incidence and prevalence remain unknown due to the condition’s rarity. However, research indicates that it predominantly manifests during childhood and adolescence, showing no preference for gender or race [2]. From a pathophysiological perspective, the condition is characterized by filaggrin overproduction, which accumulates as a dense band above the granular layer before incorporation into the mature keratin matrix. Additionally, reticular dermis fibroblasts show reduced numbers and contain abnormal dense granules in their plasma membranes, leading to elastic fiber fragmentation and reduction [8].

Significant genetic research has provided insights into the condition’s origins. In 1983, Greiner et al. suggested a potential link to Chromosome 2 through genetic mapping of 21 affected relatives, though this association remained inconclusive [9]. More recent research by Zhu et al. identified a mutation in the *CCDC91* gene on Chromosome 12p11, which codes for a Golgi–lysosome transport protein. This discovery may explain the defective elastic fiber production observed in the dermis [10]. Genetic testing to rule out *CCDC91* and other mutations would be a valuable addition to cases like this, although it was not performed in our patient due to resource constraints.

Clinically, the condition manifests as spontaneously appearing 2–4 mm papules with a translucent appearance. These papules may be umbilicated or hyperkeratotic, appearing yellowish or matching the surrounding skin color. They typically emerge on the lateral edges of palms and soles, either as isolated lesions or coalescing into cobblestone‐like plaques [11, 12]. A particularly unusual feature in our case is the unilateral distribution of the lesions. AKE is typically bilateral and relatively symmetric. Although asymmetric or unilateral presentations have been reported, they are rare. In our evaluation, no differences in occupational, mechanical, or environmental exposures between the two hands were identified to explain this unilateral presentation, highlighting an atypical variant of the disease.

Histopathologically, the condition presents with orthokeratotic hyperkeratosis, moderate acanthosis, and hypergranulosis. The most significant finding is the presence of elastorrhexis (scarce and fragmented elastic fibers) in the dermis [13]. Diagnosis typically relies on a combination of family history (though not all cases show familial involvement), clinical presentation, and characteristic histopathological findings of elastorrhexis, confirmed through special elastic fiber stains such as Verhoeff–van Gieson and orcein [1, 13].

The differential diagnosis for AKE is broad and requires careful clinicopathologic correlation, particularly focusing on the presence or absence of elastorrhexis. Closely related marginal and acral keratodermas must be explicitly contrasted; for instance, FAH closely mimics AKE clinically but lacks the hallmark dermal elastorrhexis [14]. Other acral conditions to consider include KEM (Ramos and Silva type), hereditary papulotranslucent acrokeratoderma, acrokeratoderma hereditarium punctatum, and mosaic acral keratosis [15]. Furthermore, it is essential to distinguish AKE from other conditions characterized by elastic fiber alteration, such as papular elastorrhexis, which shares fragmented elastic fibers but typically affects the trunk and extremities, as well as Buschke–Ollendorff syndrome and mid‐dermal elastolysis [16–18]. Additional clinical mimickers that should be ruled out include acrokeratosis verruciformis of Hopf, verruca plana, degenerative collagenous plaques of the hands, digital papular calcinosis, and the lichen amyloidosis variant of primary cutaneous amyloidosis [19].

The condition is characterized by a benign clinical course with no significant functional impairment or morbidity; however, there is no established definitive cure, and available treatments have limited or inconsistent efficacy [20]. Current literature emphasizes that treatment responses in AKE are generally modest and often temporary, frequently requiring maintenance therapy due to recurrence upon treatment discontinuation. Various therapeutic approaches have been attempted, including topical treatments (tretinoin, salicylic acid, and high‐concentration urea), systemic medications (methotrexate, dapsone, and corticosteroids), and physical interventions (cryosurgery and Erbium/YAG laser). However, treatment efficacy varies considerably among individuals, and recurrence upon treatment discontinuation is common [21–23]. Our regimen of calcipotriol/betamethasone and 50% urea showed limited and nondurable benefit.

In summary, AKE is a rare and challenging condition characterized by its distinct clinical presentation and histopathological features. This case underscores the significance of considering AKE in the differential diagnosis of keratotic acral lesions and emphasizes the need to definitively differentiate it from other conditions with dermal elastorrhexis. The modest response to treatment highlights the limitations of current therapeutic options. Continued documentation and research are crucial to advancing our understanding and improving management of this uncommon condition.

## Funding

The authors received no specific funding for this work.

## Ethics Statement

This study was conducted in accordance with the ethical principles of the Declaration of Helsinki.

## Consent

The patient was informed of the study and procedures and signed an informed consent form.

## Conflicts of Interest

The authors declare no conflicts of interest.

## Supporting Information

Additional supporting information can be found online in the Supporting Information section.

## Supporting information


**Supporting Information** CARE checklist (filled).

## Data Availability

The data that support the findings of this study are available from the corresponding author upon reasonable request.
